# Performance Evaluation of GPT-5, Grok 4, and DeepSeek R1 in Interpreting Complete Blood Count Reports for Hematologic Diseases: Retrospective Comparative Study

**DOI:** 10.2196/87802

**Published:** 2026-06-05

**Authors:** Xianfei Ye, Xinglun Qi, Lina Fan, Qian Yu, Suming Zhou, Chunyun Ren, Dagan Yang

**Affiliations:** 1 Department of Laboratory Medicine, The First Affiliated Hospital, Zhejiang University School of Medicine, 79 Qingchun Road, Hangzhou, Zhejiang, China, 86 0571-87236383; 2 Key Laboratory of Clinical In Vitro Diagnostic Techniques of Zhejiang Province, Hangzhou, China; 3 Institute of Laboratory Medicine, Zhejiang University, Hangzhou, China

**Keywords:** large language models, hematologic diseases, ChatGPT, Grok, DeepSeek, hallucination, artificial intelligence, AI

## Abstract

**Background:**

Large language models (LLMs) demonstrate potential in the laboratory, yet rigorous clinical evaluation remains limited. The opacity of LLM decision-making constrains their safe application in interpreting complete blood count (CBC) reports for hematologic diseases.

**Objective:**

This study aimed to conduct an exploratory evaluation of GPT-5, Grok 4, and DeepSeek R1 in interpreting real-world CBC reports, particularly their reasoning capabilities and clinical safety.

**Methods:**

This single-center retrospective study analyzed 100 CBC reports from initial-visit patients with hematologic conditions. After responses were generated by the 3 LLMs using standardized Chinese prompts, four trained laboratory physicians blindly evaluated them across 6 quality and 5 task dimensions. Interrater reliability was assessed using intraclass correlation coefficients (ICCs), and performance differences were assessed based on 4-rater consensus scores and Friedman and Wilcoxon tests. For task 4 (ablation analysis), the McNemar test was used to compare top-1 diagnostic concordance with the gold-standard diagnosis within each model, with and without initial clinical suspicion in the prompt. Error types and distributions were documented during the task evaluation.

**Results:**

DeepSeek R1 demonstrated excellent interrater reliability across most quality dimensions (ICC ≥0.75). In the quality dimension, DeepSeek R1 significantly outperformed the other models in comprehensiveness, accuracy, clarity, relevance, and practicality. In the task 4 evaluation, GPT-5 demonstrated the highest concordance (93/100, 93%) with gold-standard diagnoses, followed by DeepSeek R1 (92/100, 92%) and Grok 4 (89/100, 89%). After removing the initial clinical suspicion, these rates decreased to 79% (79/100), 77% (77/100), and 72% (72/100), representing statistically significant within-model reductions for all models (*P*<.001). Post hoc error analysis revealed distinct patterns across task dimensions. GPT-5 exhibited 12 hallucinations in the analyzer alert processing task; DeepSeek R1 demonstrated 1 hallucination in the abnormal item identification task, whereas Grok 4 displayed none. All models exhibited reasoning errors and varying degrees of deficiencies in the correlation analysis and preliminary diagnosis tasks, characterized by unwarranted inferences of disease status from isolated results without clinical integration. Grok 4 generated 9 reasoning errors in the clinical management task by providing generic recommendations not tailored to case-specific CBC data, potentially compromising individualized treatment decisions.

**Conclusions:**

While current LLMs demonstrate potential for interpreting CBC reports in hematologic diseases, they show performance heterogeneity across models. The ablation study findings underscore the necessity of integrating clinical context for accurate laboratory test interpretation. Low scores, hallucinations, and reasoning errors in model outputs indicate that current clinical deployment requires human oversight and quality control. As this single-center, Chinese-language exploratory assessment provides only preliminary, possibly context-dependent evidence, multicenter, cross-lingual prospective validation is needed to delineate the practical boundaries and safety standards for clinical deployment.

## Introduction

The rapid advancement of next-generation information technologies has enabled large language models (LLMs), exemplified by ChatGPT (OpenAI), to demonstrate unprecedented capabilities in natural language processing, logical reasoning, and content generation [1]. In laboratory medicine, LLMs have demonstrated potential utility in multiple contexts, including laboratory test ordering [2], report interpretation [3,4], intelligent question answering [5], qualification examinations [6], laboratory management [7], and clinical decision support [8].

However, existing evaluations of LLMs predominantly rely on public or simulated datasets, with limited rigorous assessments in authentic clinical environments [9]. Critical challenges—including model hallucinations, opaque decision-making processes, and inadequate evaluation frameworks—severely constrain the safe deployment and widespread adoption of LLMs in clinical practice [10]. In response, guidelines such as TRIPOD-LLM and the Chatbot Assessment Reporting Tool (CHART) have been introduced, emphasizing the necessity of conducting systematic assessments of clinical artificial intelligence (AI) tools that are multidimensional, interpretable, and transparent [11,12].

The interpretation of complete blood count (CBC) results represents a key application for hematologic disease screening. As a primary diagnostic tool, CBC analysis provides essential clues for disease identification and differential diagnosis. When combined with clinical data from electronic health records (EHRs), CBC information from laboratory analyzers constitutes the foundation for diagnosing hematologic disorders. This process relies heavily on physician experience, leading to subjectivity, high workloads, and a lack of standardization. Traditional machine learning models have shown potential—such as CatBoost models for thalassemia identification [13], extreme gradient boosting (XGBoost)–based classifiers for acute leukemia subtypes [14], and support vector machine or artificial neural network models for acute leukemia diagnosis [15,16]. However, these models often suffer from limitations including limited interpretability, weak cross-disease generalization, an inability to integrate unstructured clinical narratives, and a lack of interactive explanations [17,18].

In contrast, LLMs leverage robust contextual understanding and chain-of-thought reasoning to identify abnormal values, analyze parameter correlations, simulate clinical reasoning, and generate diagnostic recommendations [19,20]. Notably, the DeepSeek (High-Flyer) model has been deployed in nearly 1000 Chinese hospitals [21], and its report interpretation capabilities have been piloted for laboratory result verification, clinical consultation, and patient communication.

Despite these advances, clinical laboratories currently lack evidence-based criteria for selecting LLMs, comparative evaluations using real-world clinical data, and systematic analyses of critical safety issues such as model hallucinations. This study addresses these limitations by comprehensively evaluating 3 advanced LLMs—DeepSeek R1, Grok 4 (SpaceXAI), and GPT-5—using real-world clinical CBC data across 6 quality and 5 task dimensions, with particular attention to reasoning capabilities and clinical safety. Our findings aim to provide empirical evidence and practical guidance for developing more reliable and safer AI-assisted interpretation systems for hematologic disease reports.

## Methods

### Study Design

We used a comprehensive framework to systematically evaluate the performance of 3 LLMs in interpreting CBC reports for hematologic disorders. The workflow consists of four key phases: (1) selection of target CBC reports for hematologic disorders; (2) submission of structured prompts to 3 LLMs; (3) evaluation across 6 quality dimensions—comprehensiveness, accuracy, clarity, relevance, practicality, and safety; and (4) evaluation across 5 task dimensions, reflecting clinical capabilities in analyzer alert processing, abnormal item identification, correlation analysis of abnormal items, preliminary diagnosis, and clinical management.

### Ethical Considerations

This study analyzed a set of EHRs obtained from the laboratory department of The First Affiliated Hospital, Zhejiang University School of Medicine (FAHZU) between June 1, 2025, and July 7, 2025. The study protocol was approved by the institutional review board of FAHZU (IIT2025B-0629). Prior to data extraction, all records were fully deidentified by removing all direct identifiers (eg, name, date of birth, medical record number, contact information, and clinician details) and quasi-identifiers (eg, specific dates, locations, and institutional identifiers). Only data relevant to the study objectives were retained, including patient demographics, chief complaint, symptoms, physical examination findings, initial clinical suspicion, CBC reports, and alert messages generated by hematology analyzers. As the research involved a retrospective analysis of fully deidentified EHR data, the requirement for informed consent was waived by the institutional review board. No participants were contacted, and no compensation was provided.

### Selection of Clinical Case Reports

This study retrospectively screened patients with hematologic conditions presenting for their initial visit to the 4 campuses of FAHZU (Yuhang, Qingchun, Zhijiang, and Chengzhan) between June 1, 2025, and July 7, 2025, yielding an initial cohort of 449 cases. From this cohort, we selected cases based on characteristic abnormal patterns in CBC reports that could provide diagnostic clues. Cases with normal CBC results or with nonspecific or subtle presentations (eg, those seen in early lymphoma, multiple myeloma, or coagulation disorders) were excluded. Ultimately, 49% (220/449) of the cases were included. The final diagnoses in this dataset were categorized into 4 main groups as follows:

Category 1 included myeloproliferative neoplasms, characterized by the persistent clonal proliferation of specific cell lineages, such as marked leukocytosis and a full myeloid spectrum in chronic myeloid leukemia; persistently elevated hemoglobin and hematocrit in polycythemia vera; significantly increased platelet counts in essential thrombocythemia; and a characteristic leukoerythroblastic presentation in myelofibrosis.

Category 2 included acute leukemias and myelodysplastic syndromes, defined by blood cell count abnormalities and the presence of immature cells. This category includes acute myeloid leukemia and acute lymphoblastic leukemia—both marked by blasts—as well as high-risk myelodysplastic syndromes, which typically present as persistent, unexplained bicytopenia or pancytopenia, often accompanied by blasts.

Category 3 included cytopenic disorders, defined by reduced counts in one or more blood cell lineages. This category includes aplastic anemia with pancytopenia, immune thrombocytopenia with isolated thrombocytopenia, and various types of anemia characterized by specific red blood cell indices. These include microcytic hypochromic anemia in iron deficiency, macrocytic anemia in megaloblastic anemia, and microcytic hypochromic anemia in thalassemia.

Category 4 included lymphoproliferative neoplasms, characterized by clonal quantitative abnormalities in lymphocytes or plasma cells, including chronic lymphocytic leukemia with sustained absolute lymphocytosis, lymphoma with abnormal lymphocytes, and multiple myeloma, in which circulating plasma cells can be detected in some cases.

To achieve a balance between sample representativeness and evaluation workload, 100 cases were selected as the final evaluation cohort from the 220-case dataset through stratified random sampling, with disease category serving as the stratification variable.

### Selection of LLMs

Three representative LLMs were selected for evaluation in this study: the open-source DeepSeek R1 (released May 28, 2025) and the closed-source models Grok 4 (released July 10, 2025) and GPT-5 (released August 8, 2025). The model strings used were DeepSeek-R1-0528, Grok 4, and GPT-5, respectively. All models were accessed via application programming interfaces using standardized prompts, with testing conducted on August 11, 2025. To ensure consistency and reproducibility, the generation parameters were fixed at a temperature of 0.3 and a top-p value of 1.0. None of the models underwent domain-specific fine-tuning; this study was designed to evaluate their out-of-the-box performance.

### Querying LLMs

To evaluate the capabilities of LLMs in interpreting CBC reports for patients with hematologic conditions, a standardized prompt was designed. This prompt explicitly instructed the models to act as an “experienced laboratory medicine expert” and provide a professional interpretation based on the provided information. Input data encompassed patient demographics and key clinical texts from EHRs, specifically the chief complaint, physical examination findings, and initial clinical suspicion. All clinical text inputs were used in their original, unprocessed free-text format. These inputs were integrated with CBC reports containing all numerical parameters, reference intervals, and analyzer alerts. The final gold-standard diagnoses for all cases were established by clinicians according to the World Health Organization Classification (5th edition) criteria [22].

The models were required to address the following 5 tasks in sequence:

1. Analyzer alert processing. Interpret the meaning of all alert flags, assess their potential impact on result reliability, and provide specific recommendations for subsequent actions (eg, blood smear review).
2. Abnormal item identification. List all out-of-range parameters in a structured format, including values, direction of change, and brief clinical significance.
3. Correlation analysis of abnormal items. Analyze potential pathophysiological relationships among the abnormal indicators, incorporating patient demographics.
4. Preliminary diagnosis. Propose 1 to 3 of the most likely preliminary diagnoses or differential diagnoses
5. Clinical management. Provide specific, actionable suggestions regarding urgent interventions, additional tests, and follow-up.

Regarding output specifications, the models were required to respond in professional and concise Chinese, strictly follow the numbered sequence (1-5), and limit the total word count to 500 words. Any form of disclaimer was explicitly prohibited to prompt the model to make the most probable judgment. Each case query was processed in a new, isolated conversation session to prevent contextual interference between cases.

Additionally, to evaluate the independent hematologic reasoning capability of LLMs, we conducted an ablation study for task 4 by removing the initial clinical suspicion from the prompt while retaining all other patient information. Each case was thus processed under 2 conditions—with and without clinical suspicion—enabling comparison of model performance under full and blinded clinical contexts.

### Evaluation of LLM Outputs

Through random selection from all eligible personnel at the 4 campuses, we recruited 2 junior evaluators (each with 5 years of experience) and 2 senior evaluators (each with >10 years of experience). Prior to formal evaluation, all evaluators completed a standardized training program to ensure consistent application of the evaluation criteria. The training encompassed clinical practice guidelines, authoritative medical literature, and clinical experience. It also included detailed explanations of scoring dimensions, illustrative case demonstrations, and a calibration exercise involving 20 reports. This exercise was repeated until consensus was reached and a Cohen kappa (κ) coefficient of 0.7 or above was achieved.

All model outputs were standardized before evaluator review. Only plain-text final outputs were presented, and visible reasoning traces or reasoning markers were removed when present. This removal was applied only to DeepSeek R1 outputs, as the GPT-5 and Grok 4 application programming interfaces responses did not contain visible reasoning traces. The standardized outputs were then anonymized, stripped of model identifiers, and presented in randomized order to minimize evaluator recognition based on formatting or stylistic cues.

We used 2 distinct evaluation checklists. For the quality dimensions, a scoring checklist based on a 5-point Likert scale was used (Multimedia Appendix 1). For the task dimensions, a 5-point deduction rubric was tailored to each of the 5 tasks (Multimedia Appendix 2). The task-specific deduction rubric served as a guide for assigning Likert scores across the 6 quality dimensions for each task output. In cases of ambiguity, evaluators consulted relevant guidelines or literature for clarification. This evaluation generated a total of 36,000 independent quality dimension ratings, calculated as follows: 100 cases×3 models×4 evaluators×5 tasks×6 dimensions. For both the quality and task dimensions, we applied a pragmatic consensus-scoring rule, where the final consensus score was the average of the 4 evaluator ratings. In addition, the raw individual ratings were retained to facilitate distributional visualizations of rating patterns and to analyze low-score assignments by task, model, and evaluator seniority.

We assessed concordance between the LLMs’ preliminary diagnoses in task 4 and the initial clinical suspicion (under full-context conditions), as well as the final gold-standard diagnosis (under full-context and ablation conditions). For each model, paired 2×2 contingency tables were constructed based on whether the top-1 suggestion was concordant or discordant with the final gold-standard diagnosis under the 2 conditions. In addition, we classified errors identified during evaluation as either “hallucinations” (factually fabricated information) or “reasoning errors” (inferences lacking adequate clinical justification). To further emphasize clinical safety, evaluators were advised to assign a score no higher than 3 to any response containing either type of error, and all such cases were systematically recorded and reviewed in a post hoc analysis.

### Statistical Analysis

All statistical analyses were conducted using R software (version 4.3.1; R Foundation for Statistical Computing). The reliability of the averaged ratings within the junior and senior evaluator groups was estimated using the 2-way random-effects intraclass correlation coefficient (ICC) for the mean of k=2 raters (ICC [2,2]). This coefficient reflects the reliability of the mean rating derived from 2 randomly selected raters. ICC values were reported with 95% CIs and interpreted according to Cicchetti criteria [23]: poor (<0.40), fair (0.40‐0.59), good (0.60‐0.74), and excellent (≥0.75). Additionally, we calculated objective concordance metrics for task 4, including top-1 concordance with the initial clinical suspicion and top-1 concordance with the final gold-standard diagnosis under full-context and ablation conditions. To evaluate whether removal of initial clinical suspicion significantly changed diagnostic accuracy within each individual model, we compared the paired binary outcomes (concordant vs discordant with the gold-standard diagnosis) between the 2 prompt conditions using the McNemar test.

For descriptive reporting of model performance across the 6 quality dimensions, the final 4-rater consensus scores were summarized as medians and IQRs. For task-level performance, each task output was comprehensively evaluated across the 6 quality dimensions, and a task-level score was calculated by averaging the 6 dimension scores assigned by the 4 evaluators for comparative analysis. Differences among the 3 LLMs in these scores were analyzed with the Friedman test; where significant, pairwise post hoc comparisons were conducted using the Wilcoxon signed-rank test with Holm-Bonferroni correction for multiple comparisons. All tests were 2-sided, with statistical significance set at *P*<.05.

## Results

### General Characteristics

**Figure 1.**
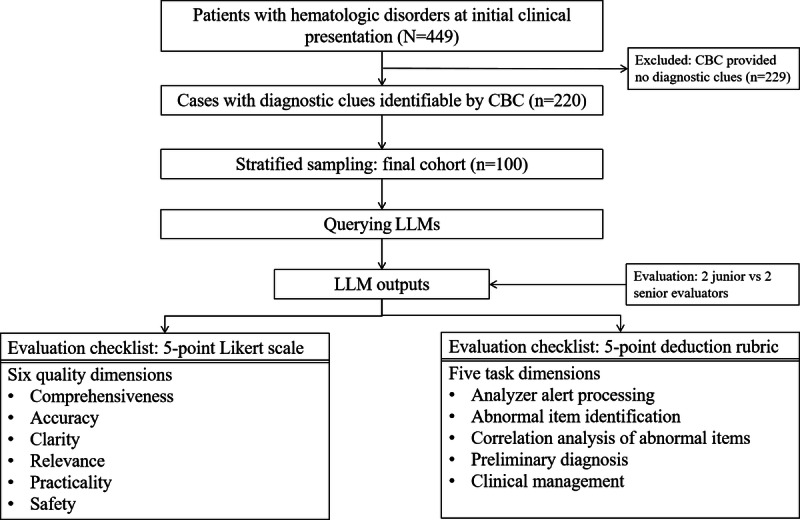
Workflow for the study design. CBC: complete blood count; LLM: large language model.

As illustrated in the study design workflow (Figure 1), after screening, exclusion, and stratified sampling across four categories of hematologic diseases, 100 patients were included in the final evaluation cohort (mean age 58.5, SD 16.9 years; range 23‐88 years; n=47, 47% male patients). The cohort composition is detailed in Table 1. The cohort encompassed diseases characterized by quantitative abnormalities in trilineage blood cell counts (category 1 and 3), as well as diseases marked by diagnostically significant pathological cells, including blasts, abnormal promyelocytes, abnormal lymphocytes, and plasma cells (category 2 and 4).

**Table 1.** Overview of clinical cases (n=100).

Characteristics	Values
Sex, n (%)	
Male	47 (47)
Female	53 (53)
Age (years), mean (SD; range)	58.5 (16.9; 23-88)
Disease category, n (%)	
Category 1: myeloproliferative neoplasms	22 (22)
Chronic myeloid leukemia	2 (2)
Polycythemia vera	5 (5)
Essential thrombocythemia	13 (13)
Myelofibrosis	2 (2)
Category 2: acute leukemias and myelodysplastic syndromes	25 (25)
Acute myeloid leukemia^a^	9 (9)
Acute lymphoblastic leukemia	6 (6)
Myelodysplastic syndromes	3 (3)
Acute leukemia of ambiguous lineage	7 (7)
** **Category 3: cytopenic disorders	34 (34)
Aplastic anemia	5 (5)
Immune thrombocytopenia	23 (23)
Iron deficiency anemia	4 (4)
Megaloblastic anemia	1 (1)
Thalassemia	1 (1)
** **Category 4: lymphoproliferative neoplasms	19 (19)
Chronic lymphocytic leukemia	12 (12)
Lymphoma with circulating abnormal lymphocytes	5 (5)
Multiple myeloma	2 (2)

a The 9 cases of acute myeloid leukemia included 2 cases of acute promyelocytic leukemia.

### Interrater Reliability

**Figure 2.**
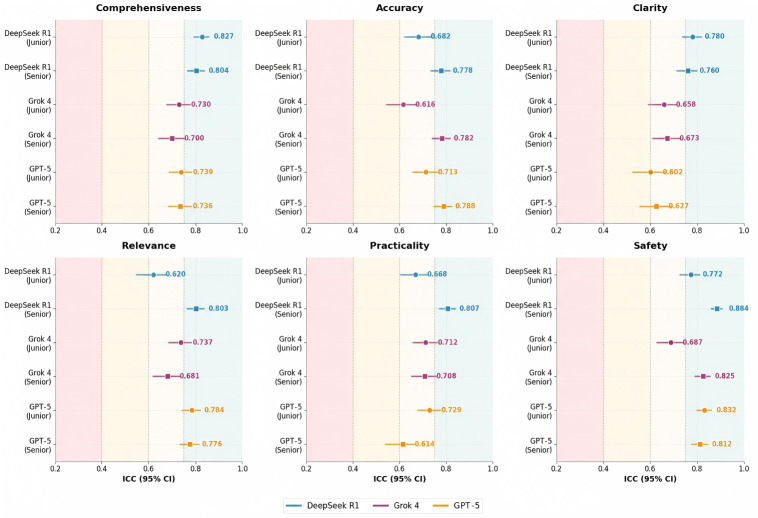
Forest plot of interrater reliability (intraclass correlation coefficient with 95% CI) for 3 large language models across 6 quality dimensions, stratified by evaluator seniority. Background colors indicate reliability levels: poor (<0.40, red), fair (0.40‐0.59, yellow), good (0.60‐0.74, light yellow), and excellent (≥0.75, light green).

Interrater reliability varied across LLMs and evaluator seniority (Figure 2). Across all 12 evaluations (2 seniority groups×6 dimensions), DeepSeek R1 demonstrated overall excellent reliability, with 9 evaluations achieving excellent reliability (ICC ≥0.75). Grok 4 showed moderate reliability, with 10 evaluations demonstrating good reliability (ICC 0.60‐0.74) and 2 achieving excellent reliability in the accuracy (ICC 0.782, 95% CI 0.740‐0.817) and safety (ICC 0.825, 95% CI 0.792‐0.853) dimensions among senior evaluators. GPT-5 exhibited relatively greater variability, with 7 evaluations showing good reliability; notably, the lowest reliability was observed in the clarity dimension among junior evaluators (ICC 0.602, 95% CI 0.525‐0.666). The remaining 5 evaluations achieved excellent reliability, primarily in the relevance and safety dimensions.

### Quality Dimension Evaluation

**Figure 3.**
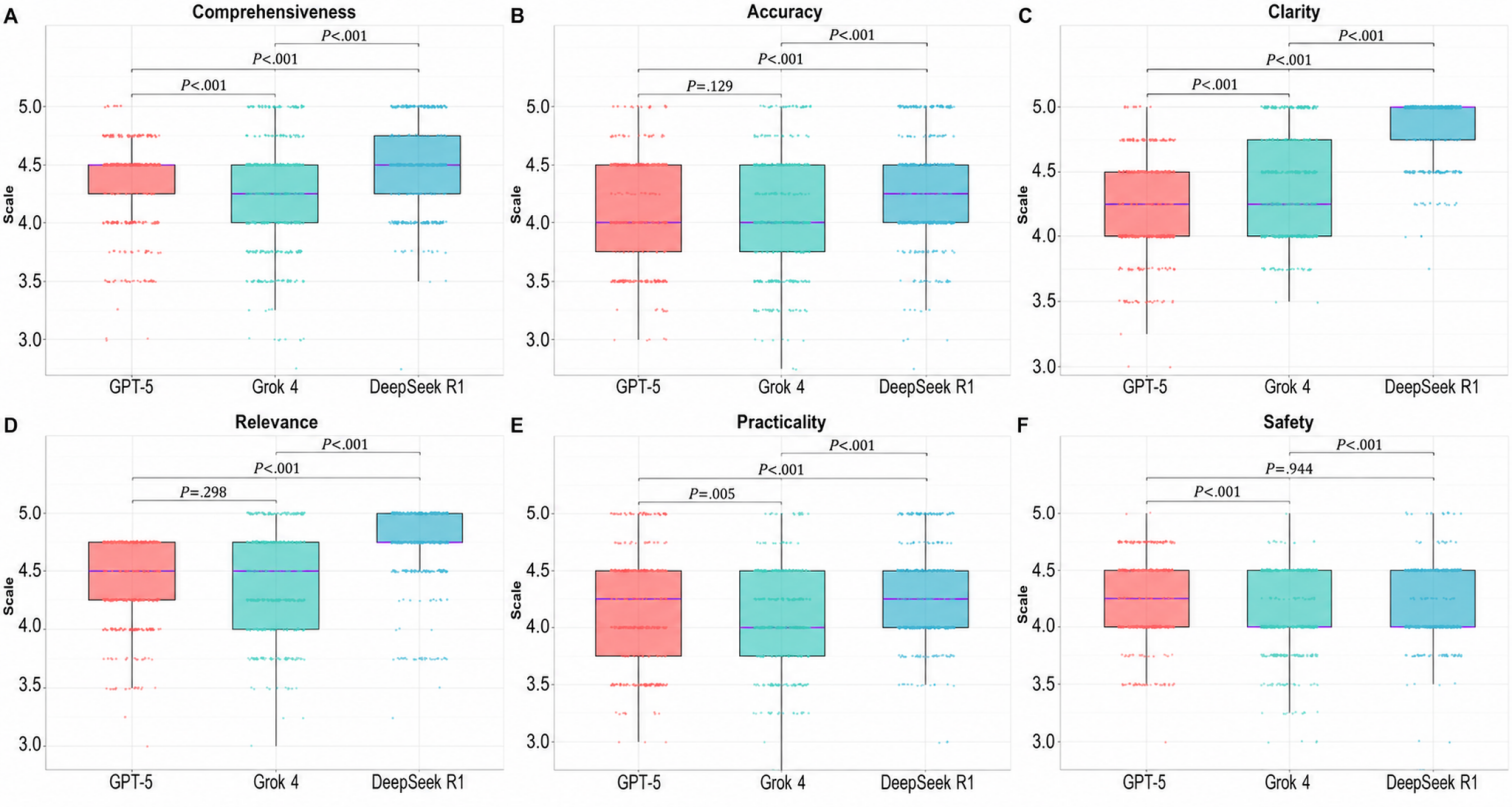
Performance comparison of GPT-5, Grok 4, and DeepSeek R1 across 6 quality dimensions. Performance is visualized using box plots, where the bounds indicate the first and third quartiles (Q1 and Q3), the internal line represents the median consensus score, and the whiskers extend to 1.5 times the IQR. Individual plotted points represent 4-rater consensus scores.

We compared the LLMs’ performance across 6 quality dimensions using box plots (Figure 3). DeepSeek R1 significantly outperformed both GPT-5 and Grok 4 in 5 dimensions: comprehensiveness, accuracy, clarity, relevance, and practicality (all *P*<.001). In the safety dimension, DeepSeek R1 achieved a median consensus score of 4.0 (IQR 4.0‐4.5), which was lower than that of GPT-5 (median consensus score 4.25, IQR 4.0‐4.5), although this difference did not reach statistical significance (*P*=.94). In comparative analyses, GPT-5 and Grok 4 demonstrated comparable accuracy and relevance, with no statistically significant differences (*P*=.13 and *P*=.30, respectively); however, GPT-5 outperformed Grok 4 in comprehensiveness, practicality, and safety (all *P*<.001). Conversely, Grok 4 exhibited significantly greater clarity than GPT-5 (*P*<.001).

### Task Dimension Evaluation

We compared the performance of the 3 LLMs across tasks 1 through 5 (Multimedia Appendix 3). DeepSeek R1 achieved the highest or tied-for-highest consensus scores in all tasks, with the only nonsignificant difference from GPT-5 occurring in the clinical management task. In the direct comparison between GPT-5 and Grok 4, the 2 models showed no significant difference in the analyzer alert processing task; furthermore, GPT-5 underperformed Grok 4 in the abnormal item identification task but outperformed it in the remaining 4 tasks.

**Figure 4.**
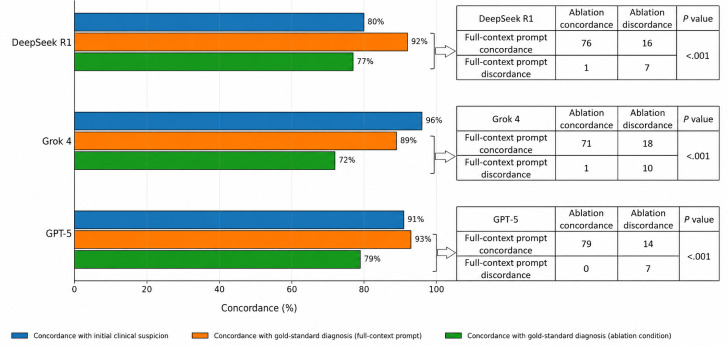
Ablation analysis of preliminary diagnosis performance in 3 large language models. Top-1 concordance rates are shown for agreement with the initial clinical suspicion, agreement with the gold-standard diagnosis under full-context prompting, and agreement with the gold-standard diagnosis under the ablation condition. Paired 2×2 contingency tables and the McNemar test were used to evaluate within-model changes after removal of initial clinical suspicion. The numbers shown in the 2×2 contingency tables represent absolute case counts out of the 100 included cases.

For task 4 (preliminary diagnosis), we compared top-1 model outputs against both the initial clinical suspicion and the final gold-standard diagnosis under full-context and ablation conditions (Figure 4). Under full-context conditions, Grok 4 showed the highest concordance with the initial clinical suspicion (96/100, 96%), whereas GPT-5 achieved the highest concordance with the gold-standard diagnosis (93/100, 93%), followed by DeepSeek R1 (92/100, 92%) and Grok 4 (89/100, 89%). After removal of initial clinical suspicion, concordance with the gold-standard diagnosis declined to 79% (79/100) for GPT-5, 77% (77/100) for DeepSeek R1, and 72% (72/100) for Grok 4. The McNemar test showed that these within-model declines were statistically significant for all 3 models (all *P*<.001), indicating that initial clinical suspicion materially improved diagnostic accuracy.

### Error Distributions and Analysis

**Figure 5.**
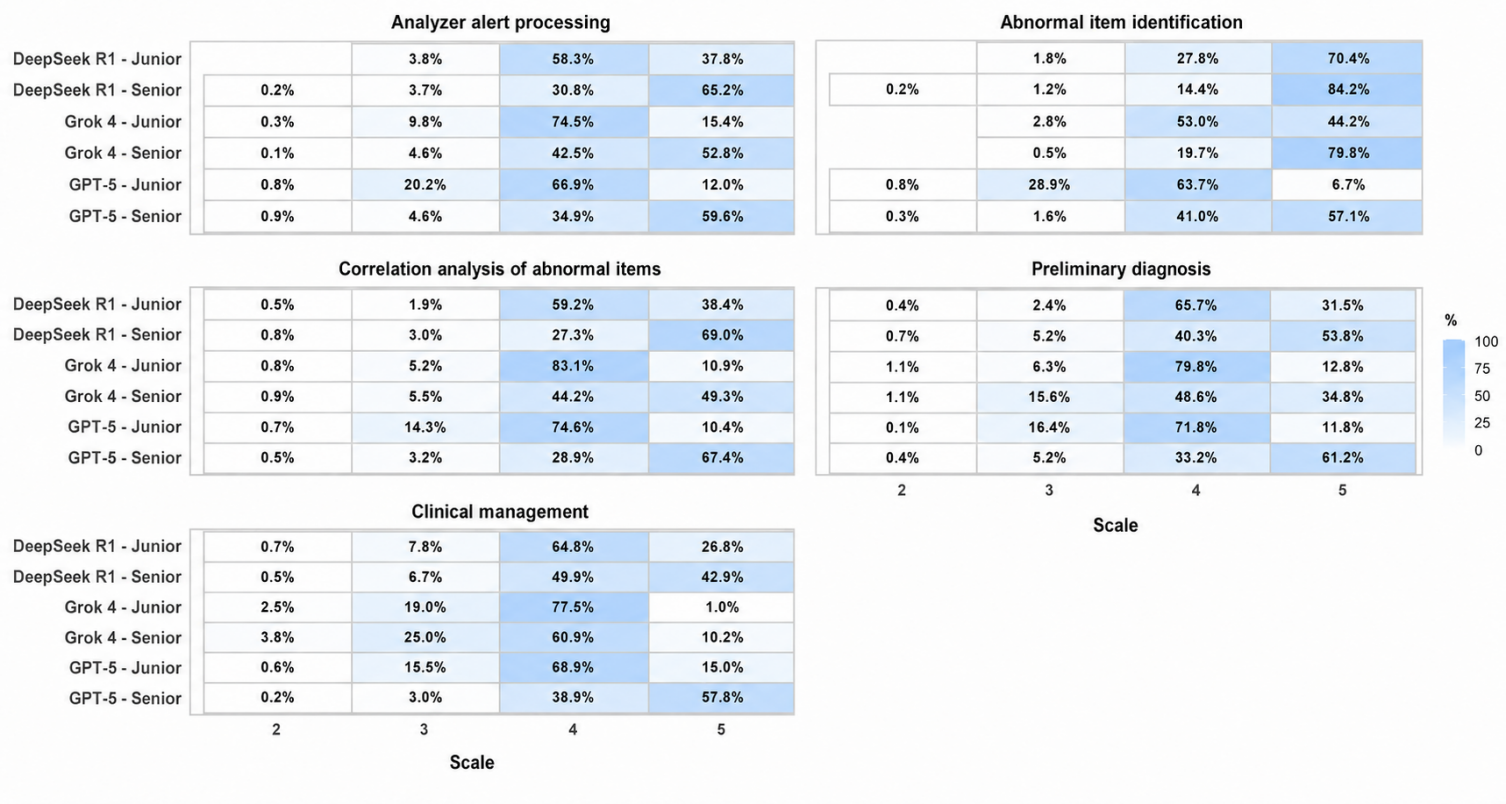
Heatmap analysis of ratings across 5 task dimensions by evaluator seniority. Score distributions (1-5) for 3 large language models are shown, stratified by junior and senior evaluators, with ratings across the 6 quality dimensions. The color gradient and numeric values in each segment represent the proportion of assignments for each score. Scores of 1 are not displayed because no evaluator assigned a rating of 1 in the study dataset.

We visualized raw individual evaluator rating distributions using heatmaps (Figure 5), with 1200 ratings generated for each task-model-evaluator seniority group combination (100 cases×2 evaluators within the seniority group×6 quality dimensions). Acknowledging that central tendency metrics (eg, median consensus scores) can obscure low-score assignments in clinically important tasks, we specifically analyzed the tails of the distribution. Although the rating scale theoretically ranged from 1 to 5, no evaluator assigned a score of 1 in this dataset; therefore, only observed score categories (2-5) are displayed. In clinical applications, the absolute incidence of serious errors is far more consequential than average performance; therefore, we quantified responses receiving low scores (<3) and qualitatively assessed the clinical risk. These low scores were mainly concentrated in 2 tasks: preliminary diagnosis and clinical management. In the preliminary diagnosis task, Grok 4 showed a relatively high proportion of low scores, at 1.1% (13/1200) for both junior and senior evaluators. In the clinical management task, the proportion of low scores for Grok 4 was even higher, at 2.5% (30/1200) for junior evaluators and 3.8% (46/1200) for senior evaluators.

To elucidate the specific errors underlying these scores, we conducted a narrative review of the error distribution across the 5 task dimensions (Multimedia Appendix 4). In the analyzer alert processing task, hallucination errors were most prominent. GPT-5 exhibited 12 such errors, including recommending the manual correction of white blood cell counts while ignoring modern analyzers’ automatic correction features, and misinterpreting plasma cell percentages from manual differential counts as instrument results—all of which could lead to unnecessary manual review and delayed reporting. DeepSeek R1 showed minor misunderstandings regarding platelet count interference factors, whereas Grok 4 displayed no hallucinations in this task.

For the abnormal item identification task, only DeepSeek R1 exhibited a single hallucination error (misinterpretation of reference interval thresholds), potentially leading to false-positive or false-negative clinical judgments. During the correlation analysis task, all models exhibited reasoning errors, characterized by unwarranted inference of disease status from a single laboratory result without integrating prior clinical information. Such errors could precipitate inappropriate clinical escalation, patient anxiety, and unnecessary overtesting. These inferential errors propagated further into the preliminary diagnosis task, where models used definitive terminology lacking guideline support to assert disease progression, potentially misguiding clinicians toward inappropriate treatment decisions. In the clinical management task, Grok 4 generated 9 reasoning errors by providing generic recommendations not tailored to specific CBC reports, which could compromise patient-specific health care and delay appropriate treatment. By comparison, DeepSeek R1 showed only 1 such case, and GPT-5 displayed none.

## Discussion

### Principal Findings

This multidimensional comparative evaluation of 3 leading LLMs in interpreting CBC reports for hematologic diseases demonstrates substantial potential for integration into laboratory medicine, offering critical insights for clinical implementation while revealing performance heterogeneity among models in handling complex hematologic inference.

Significant performance heterogeneity emerged across models in a task-dependent manner. DeepSeek R1 demonstrated superior or equivalent performance across all 6 quality dimensions and 5 clinical tasks. Its reasoning architecture—optimized through large-scale reinforcement learning and pretrained on high-quality Chinese medical literature—enabled the generation of localized and logically rigorous interpretations that garnered high evaluator recognition [24,25]. More importantly, the model exhibited substantial independent diagnostic capabilities: despite achieving only 80% (80/100) concordance with preliminary clinical suspicion, it attained 92% (92/100) concordance with gold-standard diagnoses, indicating its capacity for independent reasoning that challenges initial suspicions rather than merely reinforcing them [26].

In contrast, Grok 4 exhibited concerning error patterns in clinically critical tasks and systematic confirmation bias. While demonstrating 96% (96/100) concordance with preliminary clinical suspicion, its concordance with final gold-standard diagnoses was merely 89% (89/100), suggesting a tendency to extract and reinforce initial diagnostic cues from prompts rather than conducting independent inference based on laboratory data. Its concordance with the gold-standard diagnosis also declined markedly after ablation, from 89% (89/100) to 72% (72/100) (McNemar *P*<.001). Furthermore, it displayed systematic overdiagnosis tendencies, generating generic management recommendations lacking specific data support during clinical management tasks, with 3.8% (46/1200) of outputs receiving low scores in the senior group.

GPT-5 demonstrated overall robust performance characteristics, trailing DeepSeek R1 across most quality dimensions while retaining the highest blinded concordance after ablation at 79% (79/100). It achieved the highest concordance with gold-standard diagnoses at 93% (93/100) and with initial clinical suspicion at 91% (91/100), indicating favorable knowledge generalization and diagnostic stability. However, the model showed a unique pattern of technical hallucinations in task dimensions, with 12 factual errors in the analyzer alert processing task, indicating that even generational upgrades of general-purpose LLMs cannot eliminate knowledge gaps in specialized technical domains.

The ablation study findings underscore the importance of integrating clinical context for accurate interpretation of laboratory results, which is highly consistent with the perspective advocated by Plebani [27] on laboratory result interpretation: interpreting laboratory data in isolation from the clinical context is inherently limited and potentially misleading. Plebani [27] explicitly noted that expecting AI to achieve more accurate diagnoses than well-trained clinicians based solely on reference intervals and test parameters is “absurd,” emphasizing that true laboratory medicine value realization depends on the integration of pretest probability and comprehensive clinical information. In our study, all models showed significant declines after removal of the initial clinical suspicion, further supporting the notion that, in the absence of clinical information, models tend to rely excessively on statistical associations rather than pathophysiological reasoning. Although GPT-5 demonstrated relative robustness under blinded conditions, this cannot compensate for the systematic loss of diagnostic accuracy when removed from clinical context. Therefore, current applications of LLMs in hematology report interpretation should adhere to the principle advocated by Plebani [27] that laboratory results must be interpreted within the context of pretest probability and comprehensive clinical information, restricting AI assistance to scenarios with a complete clinical background rather than permitting its use as an isolated interpreter of test results.

### Comparison With Prior Work

Previous studies have predominantly relied on publicly available question banks or simulated cases with limited validation in real clinical scenarios, functioning essentially as “isolated test result interpretation tools” rather than “integrated clinical decision support systems.” Such research only assessed the models’ ability to interpret individual laboratory parameters without evaluating LLMs’ performance in complete diagnostic contexts [3]. For instance, Kumari et al [28] evaluated 3 LLMs on 50 complex, multitopic hematology cases to assess their case-solving performance; Han et al [29] assessed ChatGPT’s error-correction capability for nucleic acid testing reports by artificially introducing mistakes; and Cadamuro et al [3], representing the European Federation of Clinical Chemistry and Laboratory Medicine Working Group, tested ChatGPT’s comprehension using 10 simulated reports of common parameters. Additional studies compared LLMs with physician interpretations of laboratory questions from online health forums [5,19], yet remained confined to general comprehension rather than professional diagnostic reasoning. Unlike prior work, this study integrates comprehensive EHR context (chief concerns and physical examination findings) with raw analyzer data and alert flags, constructing an evaluation framework that more authentically replicates clinical laboratory workflows. This paradigm shift from “test interpretation tool” to “clinical decision support” provides a feasible pathway for transitioning laboratory report interpretation from bench to bedside deployment.

Despite the potential of LLMs, hallucinations and reasoning errors remain fundamental barriers to clinical implementation [9]. Based on our error classification framework, differentiated strategies are required for precise risk mitigation. For hallucination errors exhibited by GPT-5, retrieval-augmented generation can anchor model outputs to Clinical and Laboratory Standards Institute (CLSI) guidelines and instrument operation manuals, eliminating fabricated instructions such as “manual correction of white blood cell counts” [20,30]. For reasoning errors demonstrated by Grok 4, senior hematologists should be specifically tasked with reviewing diagnostic recommendations, leveraging their clinical reasoning to calibrate AI overinference. DeepSeek R1’s superior performance as an open-source model suggests that domain-specific fine-tuning based on local practice guidelines can significantly enhance evidence-based reasoning consistency, while future integration of multimodal inputs—such as peripheral blood smear images and CBC scattergram raw data—may further augment diagnostic reliability [9]. Based on these differentiated strategies, we recommend implementing a tiered human-AI collaboration framework: structured tasks, such as abnormal item identification, may be automated using LLMs to improve efficiency, whereas high-risk steps—including analyzer alert interpretation, preliminary diagnosis, and clinical management tasks—must undergo mandatory review by hematology experts to ensure clinical safety.

### Limitations

This study has several limitations. First, although cases from 4 hospital campuses were included, the retrospective design and stratified sampling based on typical CBC abnormality patterns may introduce selection bias, particularly by excluding diseases with subtle CBC presentations—such as early-stage lymphomas and multiple myeloma without cytopenias—resulting in an overrepresentation of classic cases. Second, the evaluation was conducted in Chinese, whereas the base training data for GPT-5 and Grok 4 are primarily in English, which may have introduced a language-related confounding factor. In addition, the prompt imposed a strict 500-word output limit, which represented another artificial constraint that may have influenced model behavior. This restriction may have encouraged models to compress content or selectively omit details to comply with the length requirement, thereby affecting the overall quality of the responses. The effects of language and length constraints may have jointly contributed to some of the observed patterns in this study, such as the relatively lower clarity of GPT-5. For Grok 4, the relatively generic and less tailored clinical management recommendations may be related to the model attempting to shorten its response to comply with the prompt instructions. For DeepSeek R1, the 500-word limit was applied only to the final output rather than to reasoning tokens; therefore, the models may not have been evaluated under fully equal length constraints, potentially confounding task performance. Future studies should consider language-matched evaluation settings and more flexible output constraints to better reflect real-world model capabilities. Third, the structured fact-verification checklist standardizes the grading format; however, in the absence of a pre-established, case-specific answer key, distinguishing between verifiable minor errors and significant interpretive deviations requires subjective clinical judgment that may vary among evaluators, leaving residual subjectivity in the rubric definitions. Future studies should develop comprehensive, case-specific scoring rubrics with predefined exemplar responses to further enhance objectivity. Fourth, only standardized final outputs were retained for evaluation, with visible reasoning traces excluded. Because this preprocessing was applied only to DeepSeek R1, which uses a reinforcement learning–optimized reasoning mechanism to generate high-quality outputs, evaluators were unable to assess its intermediate deductive process. This may have limited the evaluation of reasoning transparency and error formation mechanisms, particularly for DeepSeek R1. Fifth, the number of hematologists participating in the evaluation was limited and all were from the same health care system, which may introduce institution-specific bias and affect the generalizability of the results. Finally, given the rapid evolution of LLM technology, our results represent only a performance snapshot at a specific time point; subsequent model updates may alter performance characteristics, limiting the strict reproducibility of findings.

### Conclusions

This study reveals significant performance heterogeneity among LLMs in real-world hematologic CBC report interpretation and distinct patterns of error distribution, providing preliminary evidence for laboratory AI tool selection. Clinical deployment should implement a tiered management strategy based on error classification: restricting LLMs to low-risk structured task assistance while mandating expert review for high-risk diagnostic reasoning tasks. As this represents a single-center, Chinese-language exploratory assessment, this findings are context-dependent and necessitate multicenter, cross-lingual prospective validation to further delineate the safety boundaries and generalizable standards for clinical integration of LLMs.

## Supplementary material

10.2196/87802 Multimedia Appendix 1 Evaluation checklists for quality dimensions.

10.2196/87802 Multimedia Appendix 2 Evaluation checklists for task dimensions.

10.2196/87802 Multimedia Appendix 3 Performance comparison of GPT-5, Grok 4, and DeepSeek R1 across 5 task dimensions.

10.2196/87802 Multimedia Appendix 4 Narrative review of model errors across 5 task dimensions.

## Abbreviations

AI: artificial intelligence
CBC: complete blood count
CLSI: Clinical and Laboratory Standards Institute
EHR: electronic health record
FAHZU: The First Affiliated Hospital, Zhejiang University School of Medicine
ICC: intraclass correlation coefficient
LLM: large language model
