# Supplementary material for: Performance Evaluation of GPT-5, Grok 4, and DeepSeek R1 in Interpreting Complete Blood Count Reports for Hematologic Diseases: Retrospective Comparative Study
Source: J Med Internet Res. 2026 Jun 5;28:e87802. doi: 10.2196/87802 (PMC13240632; doi:10.2196/87802)

Multimedia Appendix 3：Performance comparison of GPT-5, Grok 4, and DeepSeek R1 across five task dimensions


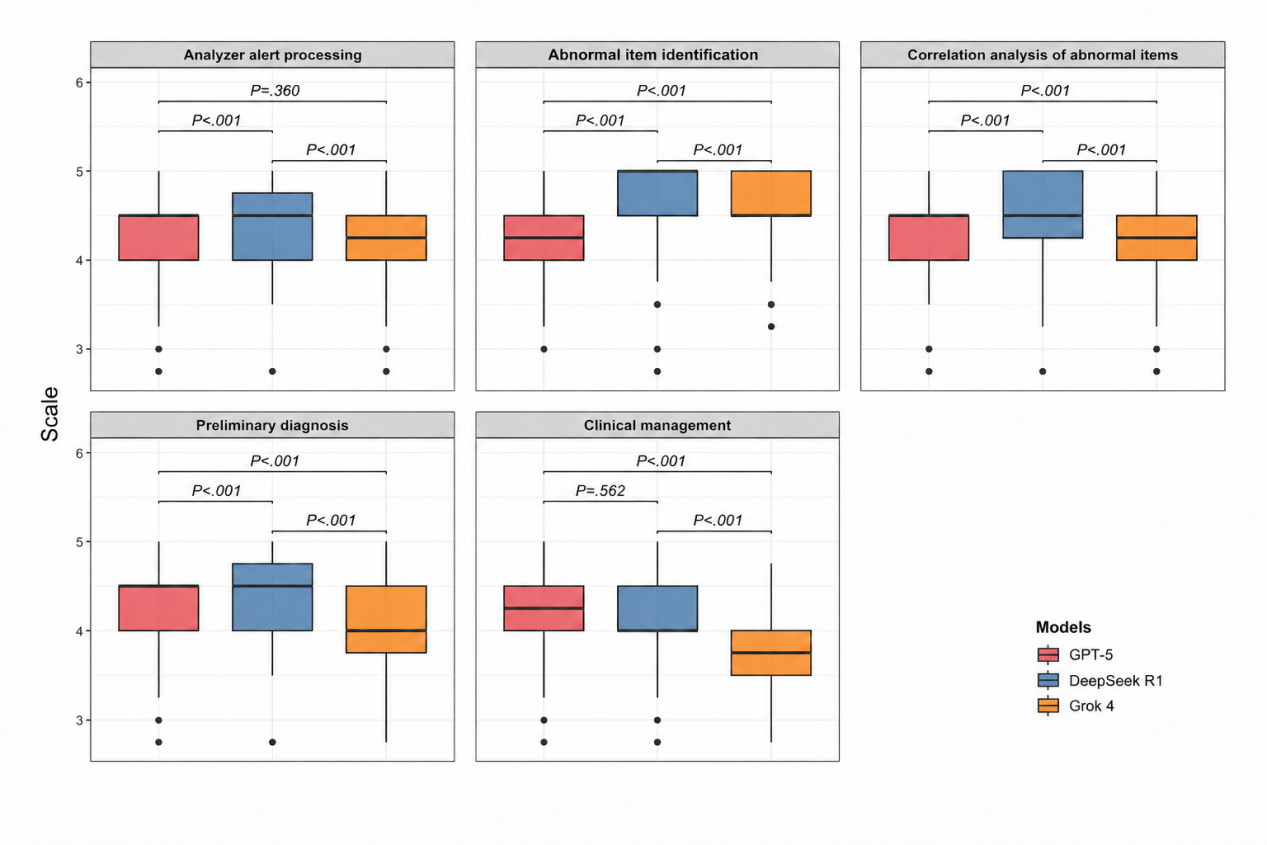

Supplement: Multimedia Appendix 3 [file jmir-v28-e87802-s003.docx]
