# Supplementary material for: Performance Evaluation of GPT-5, Grok 4, and DeepSeek R1 in Interpreting Complete Blood Count Reports for Hematologic Diseases: Retrospective Comparative Study
Source: J Med Internet Res. 2026 Jun 5;28:e87802. doi: 10.2196/87802 (PMC13240632; doi:10.2196/87802)
Supplement: Multimedia Appendix 4 [file jmir-v28-e87802-s004.docx]

**Multimedia Appendix 4** Narrative review of model errors across five task dimensions

| **Task Dimension** | **Error Type** | **Examples and Notes – GPT-5** | **Examples and Notes – Grok 4** | **Examples and Notes – DeepSeek R1** |
| --- | --- | --- | --- | --- |
| Analyzer alert processing | Hallucination | 12 cases: Suggests manual white blood cell count correction upon detection of nucleated red blood cells, failing to acknowledge current instrumental methodologies capable of automatic correction (n=10); Misinterprets a plasma cell percentage (6%) identified via manual differential as an automated instrument result and redundantly recommends manual morphological confirmation (n=2). | None found. | 1 case: Incorrectly states that cells (e.g., immature granulocytes, nucleated RBCs) flagged by instrument alerts can interfere with automated platelet count accuracy (Case 56). |
|  | Reasoning error | None found. | None found. | None found. |
| Abnormal item identification | Hallucination | None found. | None found. | 1 case: Incorrectly interprets numerical values against given reference intervals. For example, in Case 32, a hemoglobin level of 116 g/L (reference range 115–150 g/L) in a 33-year-old female was misinterpreted as mildly decreased, suggesting anemia, whereas the value is at the lower end of normal. |
|  | Reasoning error | None found. | None found. | None found. |
| Correlation analysis of abnormal items | Hallucination | None found. | None found. | None found. |
|  | Reasoning error | 7 cases: The model's correlation analysis between abnormal parameters and disease states lacks sufficient evidence and can be overly assertive. For instance, in Case 9, based solely on a single CBC showing significant lymphocytosis in a patient with an unremarkable clinical examination, the model defined the patient's CLL status as "active" or "progressive." | 6 cases: Same as GPT-5. | 6 cases: Same as GPT-5. |
| Preliminary diagnosis | Hallucination | None found. | None found. | None found. |
|  | Reasoning error | 7 cases: The model's diagnosis of CLL as being in an active, relapsed, or progressive stage is inadequately supported (see "Correlation analysis"). | 6 cases: Same as GPT-5. | 6 cases: Same as GPT-5. |
| Clinical management | Hallucination | None found. | None found. | None found. |
|  | Reasoning error | None found. | 9 cases: Provides generic therapeutic advice not tailored to specific CBC data. For example, in Case 82, with a platelet count of 32×10⁹/L and no documented bleeding symptoms, the model recommended immediate platelet transfusion without a prior clinical bleeding risk assessment. | 1 case: Suggested investigations misaligned with current clinical presentation. For example, in Case 76, for a 31-year-old male nearly one year post-ALL diagnosis without signs of neutropenia or fever, the model recommended blood cultures. |
